# Ear abnormalities in patients with oculo-auriculo-vertebral spectrum (Goldenhar syndrome)

**DOI:** 10.1590/S1808-86942011000400008

**Published:** 2015-10-19

**Authors:** Rafael Fabiano Machado Rosa, Alessandra Pawelec da Silva, Thayse Bienert Goetze, Bianca de Almeida Bier, Sheila Tamanini de Almeida, Giorgio Adriano Paskulin, Paulo Ricardo Gazzola Zen

**Affiliations:** 1 Master's degree, medical geneticist at UFCSPA/CHSCPA, and doctoral student in the graduate program on pathology, UFCSPA; 2 Community physicians, resident in the medical genetics program, UFCSPA, Brazil; 3 Speech therapy student, UFCSPA, Brazil; 4 Speech therapy student, UFCSPA, Brazil; 5 Master's degree, speech therapist, professor of the speech therapy course, UFCSPA. Doctoral student in the graduate program on gastroenterology, Rio Grande do Sul Federal University (UFRGS), Brazil; 6 Doctoral degree, medical geneticist at UFCSPA/CHSCPA. Professor of clinical genetics and of the graduate program on pathology, UFCSPA. Cytogeneticist in charge of the Laboratório de Citogenética (Cytogenetics Lab), UFCSPA, Brazil; 7 Doctoral degree, medical geneticist, UFCSPA/CHSCPA. Professor of clinical genetics and of the graduate program on pathology, UFCSPA, Brazil

**Keywords:** ear, ear auricle, ear canal, facial asymmetry, goldenhar syndrome

## Abstract

**Abstract:**

Oculo-auriculo-vertebral spectrum (OAVS) is a rare condition characterized by the involvement of the first branchial arches.

**Purpose:**

To investigate the ear abnormalities of a sample of patients with OAVS.

**Materials and methods:**

The sample consisted of 12 patients with OAVS seen at the Clinical Genetics Unit, UFCSPA/CHSCPA. The study included only patients who underwent mastoid computed tomography and with normal karyotype. We performed a review of its clinical features, giving emphasis to the ear findings.

**Results:**

Nine patients were male, the ages ranged from 1 day to 17 years. Ear abnormalities were observed in all patients and involved the external (n=12), middle (n=10) and inner ear (n=3). Microtia was the most frequent finding (n=12). The most common abnormalities of the middle ear were: opacification (n=2), displacement (n=2) and malformation of the ossicular chain. Agenesis of the internal auditory canal (n=2) was the most frequent alteration of the inner ear.

**Conclusions:**

Ear abnormalities are variable in patients with OAVS and often there is no correlation between findings in the external, middle and inner ear. The evaluation of these structures is important in the management of individuals with OAVS.

## INTRODUCTION

Branchial arch disorders comprise several developmental anomalies including the oculo-auriculo-vertebral spectrum (OAVS), also known as Goldenhar's syndrome, or hemifacial microsomy (OMIM 164210)^1^. This is a rare, complex, and phenotypically variable condition^2,^^3^. Its estimated prevalence is 1 case for every 5,600 to 26,550 births^4^, ^5^, ^6^; the condition affects males more than females (about 3:2)^2^. The OAVS may range from mild to severe forms; facial involvement is usually asymmetric, occurring mostly on the right^2,^^7^.

The origin of the OAVS is unclear, but it is a complex and heterogeneous condition. Two pathophysiologic mechanisms have been proposed for the OAVS: a reduced blood flow, and focal hemorrhage in the development region of the first and second branchial arches around 30 to 45 days of pregnancy, in the blastogenesis period^2^. These mechanisms explain the outer ear abnormalities in this spectrum, as the first branchial arch gives rise to the anterior ear primordium, and the second branchial arch originates the posterior ear primordium. Also, the outer ear canal derives from the dorsal portion of the first branchial cleft^8,^^9^. It is thought that the etiology may be related with a migration anomaly of neural crest cells. Other evidence has suggested that there are genetic determinants in some cases. A few reports have mentioned families with recessive autosomal or dominant autosomal inheritance. The literature contains several descriptions of chromosomal anomalies and gestational exposure that mimic its phenotype (thalidomide, retinoic acid, and diabetes mellitus)^2^.

Although external ear anomalies have been described in OAVS patients - to the point of being inclusion criteria - middle and especially inner ear alterations have received little attention in the literature^9^, ^10^, ^11^, ^12^, ^13^. Thus, the purpose of this study was to investigate the clinical findings in a sample of OAVS patients, focusing on ear alterations.

## MATERIAL AND METHODS

The sample comprised 12 patients with the OAVS seen at the Clinical Genetics Unit, UFCSPA/CHSCPA. The inclusion criteria were alterations in at least two of the following bodily regions: oro-cranial-facial, ocular, auricular, and vertebral, according to Strömland et al.'s criteria (2007)^12^. Only patients with normal GTG banded karyotypes (to exclude patients with chromosomal alterations that mimic the OAVS), according to the Yunis modified technique (1981)^14^, and in which computed tomography of the mastoid including middle and inner ear assessments had been done were included. All patients belonged to the sample described by Rosa et al. (2010)^15^; these authors evaluated the frequency and type of cardiac anomalies in OAVS patients.

The registries of patients were reviewed to gather clinical data and the results of diagnostic tests (focusing on ear anomalies). Fisher's exact test (bicaudate) was applied to compare the frequencies were found. The software was the PEPI program. Values of *p*<0.05 were considered statistically significant. The institutional review board approved this study (Opinion no. 581/08 of 25/01/2008).

## RESULTS

**Figure 1:**
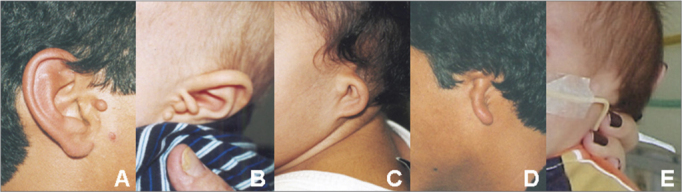
Images showing different grades of microtia in patients of the study sample: normal ear with preauricular appendage (A); grade I microtia with preauricular appendages (B); grade II microtia (C); grade Iii microtia (D), grade IV microtia or anotia (E).

**Figure 2:**
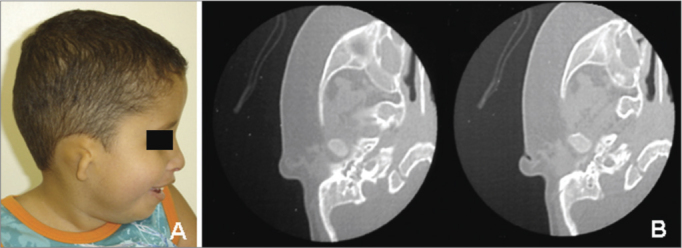
OAVS patient from the study sample (number 12 in Table 1) - the computed tomography of the mastoid showing right ear anomalies (opacified middle ear and malformed ossicular chain).

There were 9 male and 3 female subjects. The ages at the first visit ranged from 1 day to 17 years (10 were aged 1 year or less). Most of these patients were referred from the pediatrics unit (n= 7); two others were referred from the plastic surgery unit, one from the cardiology unit, one from the otorhinolaryngology unit, and one from the pediatric surgery unit. Eight subjects had two of the inclusion criteria, three subjects had three, and one had four. All subjects had ear anomalies, affecting the external ear (n= 12), the middle ear (n= 8), and the inner ear (n= 3). Microtia was the most frequent finding - it was observed in all cases (ranging from grades I to IV). It was mostly unilateral, to the left, and grade III. Four patients had bilateral microtia (33%). The most common middle ear anomaly was opacification (n= 2), and displaced (n= 2) and malformed ossicular chains (n= 2). Agenesis of the inner ear canal (n= 2) was the most frequent inner ear anomaly. The most common finding in the temporal bone was a non-aerated mastoid (n= 5). Additional alterations in other organs or systems consisted of low height (n=4), craniofacial anomalies (n= 11), ophthalmologic anomalies (n= 2), esophageal/ pulmonary anomalies (n= 6), cardiac anomalies (n= 7), abdominal anomalies (n= 4), skeletal anomalies (n= 6), and cerebral anomalies (n= 5) (Figs. 1 and 2; Table 1).

**Table 1:** Clinical findings in OARS patients in our sample

	Oculo-auriculo-vertebral spectrum (OAVS)												
Clinical findings	1	2	3	4	5	6	7	8	9	10	11	12	Total
Sex	F	F	F	M	M	M	M	M	M	M	M	M	M-9 / F-3
Ethnic group	C	M	C	C	C	M	C	C	C	C	C	C	C-2 / M-2
Age at first visit	1y	6m	2m	2d	1d	17y	2d	1m	6d	2m	5y	1m	1d - 17y
Strömland et al.'s criteria (2007)11	3	2	3	2	4	2	2	2	2	2	3	2	2-8 / 3-3 / 4-1
Low height		+						+		+	+		4
Craniofacial anomalies	+	+	+	+	+	+		+	+	+	+	+	11
Facial hypoplasia (asymmetry)	L			L	L	L		L	L		L	R	L-7 / R-1
Facial palsy				L				L		L	R		L-3 / R-1
Others	+	+	+	+	+	+			+	+	+		9
Eye anomalies			+		+								2
Ear anomalies	+	+	+	+	+	+	+	+	+	+	+	+	12
- External ear	+	+	+	+	+	+	+	+	+	+	+	+	12
Preauricular appendages	R		R		L	R	R	B	L		L		L-3 / R-4 / B-1
Microtia	+	+	+	+	+	+	+	+	+	+	+	+	12
Grade I		L	B		L				B				L-2 / B-2
Grade II				L									L-1
Grade III	L	R				L	L	L			L	R	L-5 / R-2
Grade IV										B			B-1
Agenesis of the outer ear canal	L	L		L		L	L	L		B	L		L-7 / R-1 / B-1
Stenotic outer ear canal					L		R						L-1 / R-1
- Middle ear	+	+	+			+		+		+	+	+	8
Opacified middle ear	R											R	R-2
Agenesis of the middle ear			B										B-1
Hypoplasic tympanic cleft	R												R-1
Agenesis of the tympanic cleft	L												L-1
Hypoplasic ossicular chain		L											L-1
Displaced ossicular chain								L		B			L-1 / B-1
Malformed ossicular chain											L	R	L-1 / R-1
Widened tympanic cavity						L					L		L-2
Decreased tympanic cavity								L					L-1
- Inner ear	+		+								+		3
Hypoplasic inner ear	L												L-1
Agenesis of the inner ear canal			B								L		L-1 / B-1
Altered cochlear morphology											L		L-1
Altered semicircular canals											L		L-1
Temporal bone anomalies				+				+	+	+	+		5
Non-aerated mastoid				L				B	B	B	B		L-1 / B-4
Lengthened mastoid antrum											B		B-1
Esophageal/lung anomalies				+	+		+	+		+	+		6
Congenital cardiopathies		+	+	+	+			+		+	+		7
Abdominal anomalies			+						+	+	+		4
Skeletal anomalies	+				+		+	+	+		+		6
CNS anomalies			+		+		+		+			+	5

M: male; F: female; C: Caucasian; M: mixed color; d: day(s); m: month(s); y: year(s); NA: not applicable; NE: not examined; L: left; R: right; B: bilateral.

## DISCUSSION

External ear malformations in OAVS patients ranged from slightly dysmorphic to absent ears (or anotia - the most severe form of microtia)^16^. The term microtia literally means “small ear” although in the literature it applies to small or malformed ears. These ears are often implanted lower down^17,^^18^. A few authors have suggested that microtia is the minimum form of the OAVS^8^; microtia alone or with other minor ear malformations is commonly viewed as one of the minimal criteria for diagnosing this syndrome^3,^^7,^^19^. Thus, as observed in our study (all subjects presented microtia), its frequency is high in OAVS patients (82 to 100%)^7,^^16,^^17,^^19^. In the literature, microtia has been described as being mostly unilateral and to the right^9^, which differed from our findings, where a left incidence predominated. Microtia is usually associated with the involved side of the face^16,^^17^, as shown on Table 1. Occasionally both ears may be involved (18% to 50% of cases)^7,^^16^, as seen in 33% of our study subjects. Common minor ear anomalies in these patients include preauricular appendages and pits. The former consist of skin and cartilage appendages located in any area between the tragus and the angle of the mouth. The latter consist of small depressions generally located on the anterior margin of the ascending portion of the ear helix^18^. Both may be unilateral or bilateral, and have been described in 53% to 90% of OAVS patients^16,^^19^; in our sample, 8 patients (67%) had these anomalies. Other external ear alterations were stenosis (usually in mild cases) and atresia (usually in more severe cases) of the outer ear canal (Table 1 and Frame 1).

**Frame 1:**
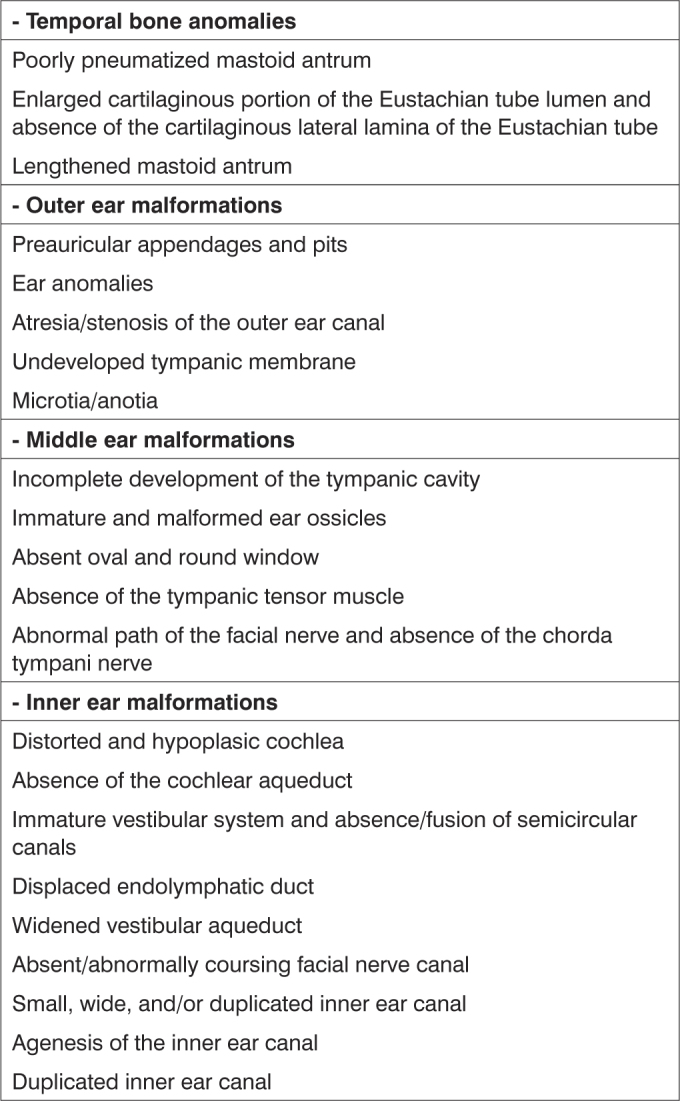
Auricular and temporal bone anomalies described in OAVS patients, according to the literature (Based on Bisdas et al., 2005^11^).

Because the ossicles of the ear develop from the dorsolateral terminations of the cartilage in the first (Meckel's cartilage) and second (Reichert's cartilage) branchial arches, anomalies of these structures are often found in the OAVS. Anomalies in the stapedial and tympanic tensor muscle suggest that these structures originate from the first and second branchial arches^9^. In our sample, 8 patients (67%) had middle ear anomalies, which is a similar to previously reported rates (75%)^12^. The main middle ear anomalies we encountered were opacification, and displaced or malformed ossicles.

Although external middle ear anomalies are well known, inner ear alterations are rarely observed; these anomalies vary widely and range from mild to severe^11^, ^12^, ^13^.

**- Temporal bone anomalies**

Poorly pneumatized mastoid antrum

Enlarged cartilaginous portion of the Eustachian tube lumen and absence of the cartilaginous lateral lamina of the Eustachian tube

Lengthened mastoid antrum

**- Outer ear malformations**

Preauricular appendages and pits

Ear anomalies

Atresia/stenosis of the outer ear canal

Undeveloped tympanic membrane

Microtia/anotia

**- Middle ear malformations**

Incomplete development of the tympanic cavity

Immature and malformed ear ossicles

Absent oval and round window

Absence of the tympanic tensor muscle

Abnormal path of the facial nerve and absence of the chorda tympani nerve

**- Inner ear malformations**

Distorted and hypoplasic cochlea

Absence of the cochlear aqueduct

Immature vestibular system and absence/fusion of semicircular canals

Displaced endolymphatic duct

Widened vestibular aqueduct

Absent/abnormally coursing facial nerve canal

Small, wide, and/or duplicated inner ear canal

Agenesis of the inner ear canal

Duplicated inner ear canal

The frequency of these anomalies in our sample (25%) was similar to previously reported rates (27% to 36%)^11,^^12^. Inner ear malformations may be agenesis of the inner ear canal, and altered cochlear and semicircular canal morphology - as seen in our study sample. Walking late in children may be associated with malformed vestibular organs, which may be seen in computed tomography^12^. Inner ear anomalies - in contrast with external and middle ear - go beyond the concept of first and second branchial arch development; in this case a neural crest cell migration disorder is an added pathogenic factor in the OAVS^9^. Stoll et al. (1998)^20^ have suggested that there are several pathogenic mechanisms causing the OAVS. Goret-Nicaise et al. (1997)^21^ have proposed that a defect in blastogenesis may explain its origin.

As mentioned above, the most frequently reported ear anomalies in the OAVS are external and middle ear abnormalities. Thus, secondary conductive hearing loss predominates in these patients^2,^^22^; the degree of hearing loss correlates directly with the level of involvement of structures^9,^^13^. However, at times a sensorineural component has been observed, which is evidenced by inner ear malformations^9,^^13^. Bilateral profound hearing loss is rare in these patients. As described in the literature, these hearing losses may cause impaired language acquisition, because speech and language develop as hearing matures^23^.

Surgery is difficult because of the complexity of this condition and the number of factors that may affect the outcome of surgical therapy. On the other hand, hearing loss is generally unilateral in the OAVS; the majority of patients develop speech, and their level of hearing enables them to be socially active. However, patients with bilateral mixed profound hearing loss or pure sensorineural hearing loss required more advanced forms of treatment, such as cochlear implants or bone anchored hearing aids^13^.

## CONCLUSION

Ear anomalies are frequent and varied in OAVS patients; often there is no correlation among external, middle, and inner ear findings. Therefore, it is important to assess these structures using radiologic methods (such as computed tomography of the mastoid) when managing patients with OAVS.

## References

[1] Online Mendelian Inheritance in Man, OMIM (TM) [homepage on the Internet]. Baltimore e Bethesda: BeMcKusick-Nathans Institute for Genetic Medicine, Johns Hopkins University and National Center for Biotechnology Information, National Library of Medicine [cited 2010 Aug 10]. Available from: http://www.ncbi.nlm.nih.gov/omim/.

[2] Cohen Jr MM, Rollinck BR, Kaye CI. (1989). Oculoauriculoveretbral spectrum: an updated critique.. Cleft Palate J..

[3] Tasse C, Böhringer S, Fischer S, Lüdecke HJ, Albrecht B, Horn D (2005). Oculo-auriculo-vertebral spectrum (OAVS): clinical evaluation and severity scoring of 53 patients and proposal for a new classification.. Eur J Med Genet..

[4] Grabb WC. (1965). The first and second branchial arch syndrome.. Plast Reconstr Surg..

[5] Stoll C, Roth MP, Dott B, Bigel T. (1984). Discordance for skeletal and cardiac defect in monozygotic twins.. Acta Genet Med Gemellol..

[6] Melnick M. (1980). The etiology of external ear malformations and its relation to abnormalities of the middle ear, inner ear and other organ systems.. Birth Defects Orig Artic Ser..

[7] Rollnick BR, Kaye CI, Nagatoshi K, Hauck W, Martin AO. (1987). Oculoauriculovertebral dysplasia and variants: phenotypic characteristic of 294 patients.. Am J Med Genet..

[8] Pearson A., English M (1978). Otolaryngology..

[9] Scholtz AW, Fish III JH, Kammen-Jolly K, Ichiki H, Hussl B, Kreczy A (2001). Goldenhar's syndrome: congenital hearing deficit of conductive or sensorineural origin? Temporal bone histopathologic study.. Otol Neurotol..

[10] Phelps PD, Lloyd GA, Poswillo DE. (1983). The ear deformities in craniofacial microsomia and oculo-auriculo-vertebral dysplasia.. J Laryngol Otol..

[11] Bisdas S, Lenarz M, Lenarz T, Becker H. (2005). Inner ear abnormalities in patients with Goldenhar syndrome.. Otol Neurotol..

[12] Strömland K, Miller M, Sjögreen L, Johansson M, Joelsson B-ME, Billstedt E (2007). Oculo-auriculo-vertebral spectrum: associated anomalies, functional deficits and possible development risk factors.. Am J Med Genet..

[13] Skarżyński H, Porowski M, Podskarbi-Fayette R. (2009). Treatment of ontological features of the oculoauriculovertebral dysplasia (Goldenhar syndrome).. Int J Pediatr Otorhinolaryngol..

[14] Yunis JJ. (1981). New chromosome techniques in the study of human neoplasia.. Hum Pathol..

[15] Rosa RFM, Dall'Agnol L, Zen PRG, Pereira VLB, Graziadio C, Paskulin GA. (2010). Espectro óculo-aurículo-vertebral e malformações cardíacas.. Rev Assoc Med Bras.

[16] Engyz O, Balel S, Unsal M, Ozer S, Oguz KK, Aktas D. (2007). 31 cases with oculoauriculovertebral dysplasia (Goldenhar syndrome): clinical, neuroradiologic, audiologic and cytogenetic findings.. Genet Couns..

[17] Touliatou V, Fryssira H, Mavrou A, Kanavakis E, Kitsiou-Tzeli S. (2006). Clinical manifestations in 17 Greek patients with Goldenhar syndrome.. Genet Couns..

[18] Carey JC., Stevenson RE, Hall JG (2006). Human malformations and related anomalies, 2e..

[19] Digilio MC, Calzolari F, Capolino R, Toscano A, Sarkozy A, de Zorzi A (2008). Congenital heart defects in patients with oculoauriculo-vertebral spectrum (Goldenhar syndrome).. Am J Med Genet..

[20] Stoll C, Viville B, Treisser A, Gasser B. (1998). A family with dominant oculoaurivertebral spectrum.. Am J Med Genet..

[21] Goret-Nicaise M, Baertz G, Saussoy P, Dhem A. (1997). Oculo-auriculovertebral spectrum: cranial and vertebral malformations due to focal disturbed chondrogenesis.. J Craniofac Genet Dev Biol.

[22] Brosco KC, Zorzetto NL, da Richieri Costa A. (2004). Perfil audiológico de indivíduos portadores da síndrome de Goldenhar.. Rev Bras Otorrinolaringol..

[23] Lima FT, de Araújo CB, Sousa EC, Chiari BM. (2007). Alterações fonoaudiológicas em um caso de síndrome de Goldenhar.. Rev Soc Bras Fonoaudiol..

